# From ‘Consent or Anonymise’ to ‘Share and Protect’: Facilitating Access to Surplus Tissue for Research Whilst Safeguarding Donor Interests

**DOI:** 10.1007/s10728-021-00435-z

**Published:** 2021-07-14

**Authors:** Catherine Blewett

**Affiliations:** Department of Law, University of Manchester, Manchester, UK

**Keywords:** Tissue, Research, Consent, Identifiability, Anonymisation

## Abstract

There is significant research value in the secondary use of surplus human tissue which has been removed during clinical care and is stored in diagnostic archives. However, this value is limited without access to information about the person from whom the tissue was removed. As the research value of surplus tissue is often not realised until after the patient’s episode of care, it is often the case that no consent has been given for any surplus tissue to be used for research purposes. The Human Tissue Act 2004 does permit research use of surplus tissue without consent, but the researcher must not be in possession of information which could identify the person from whom the tissue was removed. Due to the commonly applied ‘consent or anonymise’ approach, linking tissue and data is challenging and full anonymisation would likely render much research on surplus tissue ineffectual. This article suggests that in recognising the value in surplus tissue linked with information about the person, a ‘share and protect’ approach which considers safeguards other than anonymisation, where obtaining consent for research use would not be feasible, would better balance the public benefit of health research with the protection of individual rights and interests than a requirement for *either* consent *or* anonymisation.

## Introduction

Health research is generally considered to be something which is a ‘good’ and in the best interest of society [1], as the knowledge which is generated from research positively impacts on all members of society, whether directly or indirectly [2]. It is fundamental to the prevention, diagnosis and treatment of health-impacting conditions and, in some cases, ensures patients can have access to novel treatments which may have positive life-changing, extending or even saving effects [3]. Moreover, it can also have a positive socio-economic impact by improving the efficiency of NHS services [3]. As much health research is publicly funded however, whether this is via the Government or charities, there is also a responsibility to ensure cost efficiency in research [3]. The promotion and facilitation of efficient healthcare research is therefore considered to be something which is in the public interest [3], yet the requirement to protect the rights and interests of research participants must also play a key role and is enshrined into research ethics practice, most notably by virtue of the Declaration of Helsinki [4]. Whilst the Declaration of Helsinki is not in itself legally binding, it does provide ethical principles which are considered to have primacy and are intrinsically embedded in ethical, and to some degree legal, standards [5].

Human tissue is routinely removed from patients in the course of diagnosis and treatment. Surgical procedures such as tumour excision or biopsies [6] often involve removing relatively large amounts of tissue after which only a small amount is required for diagnostic purposes [7]. The remainder is stored in a diagnostic archive in case further testing should be required [8]. Such ‘surplus tissue’ may have value for research purposes and these diagnostic archives can be a rich source of tissue samples for such purposes [9]. Accessing existing surplus tissue samples also means that new tissue samples may not always need to be obtained. This reduces the resource required and is therefore more cost effective for the NHS [3]. However, the real value in human tissue for health research purposes comes when the tissue is linked with information about the person from whom the tissue came; the tissue alone has limited value [10]. Linking tissue with information about the person allows insight into complex interfaces between health, lifestyle, environment and genes which would be impossible from tissue alone [11], thereby strengthening the validity and utility of research involving tissue [12]. This does in turn however create further issues relating to data protection and confidentiality as linkage with information about the person can increase the possibility that the tissue may be considered ‘identifiable’.^1^ This may be due to the identifiability of the information itself, such as demographic information, or could be the linkage of information which is considered non-identifiable, but when multiple items of such information are linked together, could mean it is it possible for the person to be identified [13], such as can occur when linking multiple datasets of information [14].

The Human Tissue Act 2004 (HT Act 2004) includes research as a ‘scheduled purpose’ which means that research involving human tissue is something that can be undertaken lawfully with the consent of the person from whom the tissue was removed. However, consent for research use of tissue is often not obtained at the time of removal and it is often not feasible to obtain the person’s consent at a later point in time once the research value of surplus tissue in diagnostic archives is identified [15]. Where consent has not been provided for tissue to be used for research purposes, the HT Act 2004, permits use only where the tissue has been removed from a living person, the research has been approved by an authorised Research Ethics Committee and the researcher is not in possession, and will not likely come into possession, of information which could identify the individual from whom the tissue was removed. However, this absolute requirement for the researcher to not be in possession of information which could identify the individual does not accommodate the possibility that information which may be considered ‘identifiable’ may be crucial to be able to achieve a research aim. For example, information such as whether the tissue provider is male or female, when and where they were born, or where they currently live may all be important information to consider alongside analysis of the tissue and other clinical information in answering a research question [16]. The current application of the HT Act 2004 with regards to the researcher not being in possession of information which *could* identify the person makes availability of such data a grey area for researchers and data controllers [16]. This is because this type of information, whilst not overtly in itself identifiable, may mean that an individual could be identified when it is collated together, particularly when you consider the possibility of linking this information with other, publicly available, information such as the electoral register [16].

Whilst identifying the person may take the efforts of a ‘motivated intruder’,^2^ such a grey area in the law often leads to over caution. Even the mere possibility that re-identification could be successful may be cause for data controllers not providing information for research purposes, even when the overall research purpose could not be achieved without the information [3]. Moreover, due to the nature of research which involves surplus tissue and patient information, it may be necessary to source tissue and data from different locations as the patient may have undergone different parts of their care pathway at different NHS hospital trusts. This requires information which is unique to the individual, such as an NHS number, and often a second identifier such as date of birth to verify that the tissue and data relate to the correct individual [16]. Therefore, where consent cannot reasonably be obtained retrospectively and where certain information is necessary to achieve the research aim, the value and utility of surplus tissue is limited due to the possibility of identifiability.

This article considers an approach which aims to enable the sharing of surplus tissue where consent was not obtained at the time the tissue was removed and it would not be feasible to obtain consent retrospectively. However, it should be noted that this article does not suggest that this approach should be an *alternative* to obtaining consent where it is reasonable and feasible to do so. Consent for the future research use of tissue samples is important because it demonstrates respect for persons [17] and it allows some degree of choice [18] and control over what happens to those samples. In recent years there has been significant development in approaches to consent for the use of tissue and data in research which is laudable. For example, dynamic consent approaches using electronic platforms which can attach and send consent preferences with tissue and data when transferred from biobanks, thereby allowing greater choice with regards to the types of research which an individual permits their samples to be used for [19]. Moreover, even greater choice is afforded via a meta consent approach which also uses an electronic platform but allows individuals to choose the type of consent which they prefer; whether this is broad consent for research within defined parameters, the opportunity to consent to each individual project or blanket consent which permits any research use [20]. This article does not question the value of consent or the value of engaging with individuals about the potential research value of their surplus tissue samples and data. This article does however suggest that where tissue is removed as part of a clinically directed procedure and is surplus to diagnostic requirements, the tissue linked with associated patient data may have a future research value and such secondary uses should not be prevented *because* consent was not requested and would no longer be feasible to obtain. Moreover, this article suggests that in such situations, an approach which safeguards privacy interests by applying data protection mechanisms other than a requirement for anonymisation could be more enabling of health research which is in the public interest.

Empirical evidence suggests that generally people are supporting of their surplus tissue samples [21] and associated data being used for research purposes [22] and furthermore, they welcome the opportunity for their tissue samples to have the potential to help others [23]. In some cases, patients have questioned why this doesn’t happen more, due to the minimal impact on the tissue donor compared to the potential benefit which can be gained [24]. However, such altruistic expressions are couched within an expectation that privacy interests will be protected [25]. Moreover, the requirement for the interests of science and society to never outweigh the interests of individuals is the bedrock of research ethics and firmly rooted within the principles of the Declaration of Helsinki.^3^ Whilst research involving surplus tissue linked with associated patient data is generally considered to involve minimal risk, it is not entirely risk free [26]. For example, where tissue samples are stored in a diagnostic archive, it is important that sufficient tissue remains for any further analysis required as part of the patients clinical care [27], as well as a risk that tissue samples may be used in research which the individual would find morally reprehensible [28]. These risks can be mitigated via governance mechanisms such as access procedures and committees which consider request to access tissue samples, as well as research ethics committees which ensure that research projects are using tissue samples for ethical purposes. These risks, in the context of any mitigating governance mechanisms, need to be balanced against the potential benefit for society by undertaking health research. This article focuses on risks which are associated with data privacy, which may occur when tissue samples are linked with associated patient data, and considers how an approach which focuses on safeguarding the patient’s identity using risk mitigating mechanisms other than complete anonymisation, could better facilitate the use of tissue samples linked with patient data in health research.

## Share and protect

In this article I put forward an argument that, rather than apply a blanket requirement for non-identifiability where obtaining consent is not feasible, the use of surplus tissue in research should follow a more pragmatic approach which allows consideration of the data necessary to achieve a research aim and also respects the rights and interests of individuals by applying ‘appropriate safeguards’ to protect personal data. I refer to this approach as ‘share and protect’ because in recognising the potential benefit of combining surplus tissue and data about the person for research purposes, it considers how these resources could be shared with researchers in the public interest, whilst also requiring that there are appropriate data security measures in place which protect personal data, thereby respecting individual rights and interests.

I suggest that the origin of the requirement for non-identifiability in the absence of consent in the HT Act 2004 was based on a misperception that data protection legislation *required* that personal data should not be processed without consent and therefore the only alternative was to anonymise data where there was no consent for research use [29]. This ‘consent-or-anonymise’ approach was included in the HT Act 2004 but was not a requirement under the Data Protection Act 1998, which was in force when the HT Act 2004 was implemented. Furthermore, it is not required by the General Data Protection Regulation 2018 (GDPR 2018) or the Data Protection Act 2018 (DPA 2018) which are currently in force in the UK.^4^ Whilst ‘consent or anonymise’ may have been one way to interpret what was required under the DP Act 1998, this was not the only legitimate, or even the most appropriate interpretation [30]. The DPA 1998, and the subsequent GDPR 2018 and DPA 2018, permit data to be processed under a legal basis other than consent [13]; something which is discussed in more detail later in this article. Moreover, these legislative provisions do not require anonymisation as an alternative but rather set a requirement for ‘appropriate safeguards’ to be in place to respect the principle of data minimisation; that data should be’adequate, relevant and limited to what is necessary in relation to the purposes for which they are processed’.

I therefore argue that this interpretation of data protection legislation which facilitates sharing of the minimum data necessary to achieve a research aim, without a *requirement* for consent from the person about whom the data relates or anonymisation of the data where this would render the research ineffectual should also be applied when sharing surplus tissue linked with data about the person for health research purposes. This is because research involving surplus tissue linked with data about the person has the potential for significant public benefit but, due to the nature of surplus tissue and previously collected data, the opportunity to obtain consent for research use has often passed. Furthermore, anonymisation of the data can reduce its value and even render it ineffectual to achieve the research aim [22] and therefore a more pragmatic ‘share and protect’ approach may better achieve the balance between facilitating health research in the public interest and respecting individual rights and interests.

## Background to Research Under the Human Tissue Act 2004

To determine whether a ‘share and protect’ approach could be applied for the use of surplus tissue and data about the person from whom the tissue was removed, in the absence of consent for research use, it is important to understand how the requirement for non-identifiability to the researcher came about in the HT Act 2004. The HT Act 2004, which was a direct response to events which had been highlighted via the Kennedy and Redfern reports into practices of post mortem organ and tissue retention at Bristol Royal Infirmary and Alder Hey Hospital, aimed to balance the expectations and rights of individuals and families with broader societal interests, including health research.^5^ When the Human Tissue Bill was passing through Parliament there was significant discussion about the need to balance the importance of human tissue being available for research and, also to respect and protect the rights and interests of individuals.^6^ This balance was seemingly achieved by ensuring that the golden thread which was to run throughout the Act was consent [28]. However, a clause was introduced to the HT Act 2004 via an amendment^7^ which allows the use of surplus tissue in research without consent under certain circumstances. This amendment was in response to lobbying from the scientific research community over concern that the Act would stifle or criminalise important research.^8^ This approach acknowledged that the opportunity to obtain consent for the use of surplus tissue obtained during routine clinical procedures has often passed, particularly where the research value of the tissue is not known at the time of removal, and that obtaining retrospective consent for individual research projects is often impractical and may even be considered unethical; for example the tissue provider may have moved away, or re-contacting a person under such circumstances could bring back difficult memories of a time when they were unwell [28]. The final wording of the Act did therefore recognise that where tissue has previously been collected during the course of routine clinical care and where consent was not obtained for the tissue to be used for research purposes, it may still be used under certain conditions. The conditions are that the tissue must have been removed from a living person, the research is approved by an authorised Research Ethics Committee and the person undertaking the research must not be in possession and will not likely come into possession of information which could identify the person from whom the tissue came. However, as previously indicated, the tissue alone has limited value in a research context and will often need to be linked with information about the tissue donor. Moreover, data about the person will often be generated from tissue in the course of research and therefore tissue and data become inextricably linked.

Human tissue and data are however regulated under different statutory legislation; tissue under the HT Act 2004 and data under the GDPR 2018 and DPA 2018. The GDPR 2018 explicitly references data which is derived from the testing of body parts or bodily substances as personal data concerning health, thereby recognising the relationship between human tissue and personal data about the person from whom the tissue was removed. However, this relationship between tissue and data is not as clearly defined from the perspective of the HT Act 2004 which does not appear to accommodate the complexities of data and more importantly, the complexities of identifiability of data. Identifiability is not something that is static but rather is something that can change as more information becomes available and is a spectrum which runs from fully anonymised to fully identifiable with many levels in-between [16]. In referring only to ‘the researcher not being in possession of information which could identify the person’, the HT Act 2004 sets a rather absolute standard of non-identifiability which does not necessarily allow the flexibility necessary to deal with the complexities of data and identifiability in a research context. Not only can this approach be unduly limiting in terms of accessing surplus tissue and the information necessary to achieve a research aim, its introduction into the Act appears to have been based on a misperception that either consent from the data subject or anonymisation of data was a requirement under data protection legislation. This, as I will go on to demonstrate below, was not in fact the case.

## ‘Consent or anonymise’—A misinterpretation of data protection law?

As I have previously suggested, the HT Act 2004 applies an absolute rule of non-identifiability to the researcher for surplus tissue where there is no consent for research use. This does not acknowledge the complexity of identifiability in that identifiability can change when new information becomes available and runs a spectrum from fully anonymous to fully identifiable. I suggest that this approach was implemented due to a misperception that anonymisation was a *requirement* under data protection legislation where there is no consent for research use. In this section I will justify this claim and set out how this misperception resulted in the ‘consent or anonymise’ approach being included in the HTA Act 2004.

At the time the Human Tissue Bill (HT Bill) was drafted and discussed in Parliament, the EU Directive 95/46/EC and the Data Protection Act 1998 (DPA 1998) (which was the statute enacting the Directive into UK law) were in force. Parliamentary discussions and the final text of the HT Act 2004 do reflect a relationship between these different legislative provisions. For example, the terminology used in the HT Act, ‘not in possession, and not likely to come into possession, of information from which the person from whose body the material has come can be identified’ is derived from terminology used in the DPA 1998 which defines personal data as data from which a person can be identified……….. ‘from those data and other information which is in the possession of, or is likely to come into the possession of, the data controller’. Moreover, reference was explicitly made during the HT Bill debates in Parliament to anonymization being necessary to meet the requirements of the DPA 1998.^9^ However, such reference to anonymization in this context appears to assume the ‘consent or anonymise’ rhetoric, which was commonly interpreted as a requirement under the DPA 1998 [29, 31]. This led to an apparent perception amongst those debating the HT Bill that the only legal basis under which personal data could be processed for research purposes was where the data subject had given consent for their data to be processed for this purpose and where no consent had been given, the only alternative was to fully anonymise the data.

In referring to a requirement for ‘anonymisation’, there were numerous calls in Parliament to better define what this meant and, in particular, whether this would mean that tissue and data would be permanently unlinked. Whilst clarification was provided by Lord Warner, Parliamentary Under Secretary in the Department of Health, that a pseudoanonymised link could be maintained, a clear description of what was meant by ‘not identifiable to the researcher’, remained elusive. Lord Warner simply said that “there is no reason that the researcher should also know the name of the person that the tissue has come from”^10^. This does not address the issue of partially identifiable information which may be required to achieve a defined research aim or the potential need for a unique identifier, such as an NHS number, to link data and tissue from multiple sources. Therefore, on the understanding of the requirement for ‘consent or anonymise’ and in the absence of acknowledgement that partially identifiable information may need to be linked with surplus tissue to achieve a stated research aim, the principle of consent or anonymise was, to all intents and purposes, reflected in the HT Act 2004 [32]. However, according to the Information Commissioner’s Office, the interpretation of consent or anonymise being a requirement under the DPA 1998 was not correct and was often applied due to over caution and a desire to prevent any risk of litigation [31]. Whilst a requirement to either obtain the consent of the person or ensure that data are only processed in an anonymised format was one interpretation of the legislation, it was not the only legitimate interpretation [31] and personal data could be processed for research purposes under legal bases other than consent without a requirement for the data to be anonymised. This was permissible under the DPA 1998 due to what is often referred to as the ‘research exemption’, which permitted the use of personal data for research purposes where the data were fairly obtained, the data were not used to make decisions about the individuals, use of the data would not cause significant damage or distress and no identifiable data are published [33].

So far in this section I have suggested that the wording in the HT Act 2004 requiring for tissue to be non-identifiable to the researcher where there is no consent for research use was based on a misperception that this was required under the DPA 1998. However, as the applicable data protection legislation has changed since the HT Act 2004 was enacted, I will now also consider the position under current legislation, the GDPR 2018 and DPA 2018. The GDPR 2018 requires that for processing of personal data, including health data, a basis under both Articles 6 and 9 of its provisions must apply. Moreover, the GDPR 2018 also states that where personal data have been collected for other purposes, such as when accessing NHS services, any secondary processing of this data would be compatible with the original purpose, then the legal basis may remain the same. The GDPR 2018 is explicit that research should be considered a purpose which is compatible with the purpose for which data were collected, therefore the legal basis under which the personal data were collected may remain the same legal basis under which the data can also be processed for secondary research purposes. As confirmed by the NHS England privacy notice [34], the legal basis under the Article 6, for data being collected as part of interactions with the NHS, is not consent but is ‘processing is necessary to perform a task in the public interest’.^11^ Moreover, the legal basis under Article 9, as health data is special category data, is ‘processing is necessary for the purposes of ……… the provision of health or social care or treatment or the management of health and social care systems and services….’.^12^ Therefore, consent is not *required* as a legal basis for secondary processing of such data for research purposes. This presumption of compatibility for secondary processing of data for scientific research is however a relaxation from the DPA 1998, which required compatibility to be demonstrated [35]. Furthermore, the UK policy position, confirmed by guidance published by the Health Research Authority [36] and the Information Commissioners Officer [37] is that where personal data are processed for health research purposes, consent should in fact *not* be the legal basis used [35]. The reason for this is that the requirements in relation to consent under the GDPR 2018 are more stringent and give greater control to the data subject than the requirements which are reasonably and normatively applied in a scientific research context [35]. For consent to be valid under the GDPR 2018, it must be demonstrable, freely given, clear, in an easily accessible format, and it should be as easy to withdraw any consent given as it was to have given the consent.^13^ Whilst these standards *may* also apply in a research context, using consent as the legal basis means that these standards *must* apply for the processing to be lawful. Moreover, a broad consent approach, which is often applied for the research use of existing collections of tissue and data, would not meet the standards required for consent under the GDPR 2018 [35]. Recital 33 does acknowledge that for scientific research, the full purpose of processing personal data may not be known at the time of collection and therefore data subjects may consent to certain parts of research. However, the Article 29 working party (now the European Data Protection Board) issued official guidance which states that when relying on consent as the legal basis to process special category data, which includes health related data,^14^ the need for consent to be specific would still apply [38] and there would be an expectation that consent would continue to be sought as the research advances [39].

Whilst consent may not be required as a legal basis for the secondary processing of personal health data for research purposes under the GDPR 2018, there remains a question regarding whether anonymisation of the data would therefore be required as an alternative under this statutory provision. The GDPR 2018 refers to requirements to ensure that there are ‘appropriate safeguards’ in place to protect the rights and freedoms of the data subject. These appropriate safeguards are expected to ensure that there are technical and organisational measures in place, such as contractual arrangements to minimise who can access data and suitable security measures to prevent unauthorised access [30]. Furthermore, these safeguards should ensure that the principle of data minimisation is respected; therefore, data should be ‘adequate, relevant and limited to what is necessary in relation to the purposes for which they are processed’. The requirement for appropriate safeguards under the GDPR 2018 therefore applies a broader approach than the blanket requirement for non-identifiability under the HT Act 2004. In requiring appropriate safeguards to be in place which respect the principle of data minimisation, consideration can be given to the data *necessary* to achieve the research aim and can take a more pragmatic approach in relation to data security which may permit some identifiable data to be retained for a minimum period necessary in relation to the purpose for which it is being processed. Therefore, unlike the HT Act 2004 which is explicit that tissue which is identifiable to the researcher may only be used with appropriate consent, previously the DPA 1998 and currently the GDPR 2018 permit a lawful basis other than consent and give a broader consideration of how individual rights and interests can be protected via safeguards, other than a blanket requirement for anonymization. This therefore allows a more considered approach which takes necessity and purpose into account.

## Balancing Public Benefit with Individual Interests

So far in this article I have suggested that research involving human tissue linked with information about the person from whom the tissue was removed is in the public interest as it is cost efficient and can generate valuable data. Empirical studies have indicated that patients are not only supporting of their surplus tissue and data being used for health research purposes [23, 40], this is often their preference and they are surprised to discover that this doesn’t happen more often [21]. Moreover, patients who have the opportunity to donate surplus tissue samples for health research purposes have reported that their decision to donate was often based on a feeling of solidarity and desire to help others who may be going through a similar experience [41]. Patients have also reported that feeling solidarity with others by donating their surplus tissue for use in health research had a positive impact on their recovery [40]. Whilst patients may not necessarily be aware of individual research uses of previously removed tissue samples which were stored in a diagnostic archive, a general awareness of the potential for research to be undertaken which could benefit other patients may in itself provide some benefit for patients when undergoing investigation or treatment which involves the removal of tissue. However, the public benefit and potential for any individual benefit based on a principle of solidarity, do not negate the need for individual privacy interests to be respected.

In this article I have noted that data protection legislation does permit the processing of personal data, including health data, under legal bases other than consent and applies safeguards which are broader than just anonymisation. In this section I will set out why a similar ‘share and protect’ approach for surplus tissue and associated information would better achieve a balance between facilitating research which has public benefit and protecting the rights and interests of individuals than the current ‘consent or anonymise’ approach. The HT Act 2004, the GDPR 2018 and DPA 2018 all aim to balance the interests of individuals with public interests, such as the public benefit which can come from research. Health research generates generalisable knowledge which can have a positive impact on an individual and also societal level [3] and therefore there is a broad interest in achieving an effective balance between respecting the integrity of the individual and improving public health and minimising risks to peoples’ health [42]. The requirement for tissue to be non-identifiable to the researcher in the absence of the provider’s consent, whilst still permitting use of surplus tissue for research purposes, was intended to be a way of achieving such a balance. However, I suggest that the current application of the requirement for the person undertaking the research to not be in possession of information which *could* identify the person from whom the tissue was removed is too restrictive and therefore does not achieve this balance effectively. This is because in applying the ‘consent or anonymise’ approach it only considers two key questions: firstly, is there consent for research use? and secondly, will the researcher be in possession of information which *could* identify the person? If the answer to the first question is ‘no’, there is no consent in place, then any answer other than ‘no’ to the second question would mean that the research cannot proceed. There is no consideration of the potential for public benefit, what the research involves and what data would be required to achieve the intended aim or what other appropriate safeguards could be in place to protect the interests of the individuals.

If we apply the alternative ‘share and protect’ interpretation, which does consider the data required to achieve the research aim, then this may increase the scope within which surplus tissue and information about the person from whom the tissue was removed can be used for research purposes. This could therefore increase the research being undertaken and thereby also increase the net utility generated from such research. This is because the ‘share and protect’ approach considers the same scenario from a different perspective. Instead of an assumption that surplus tissue and data cannot be shared because there is no consent in place, the ‘share and protect’ approach accepts that there is benefit in sharing tissue and data for research purposes but considers what safeguards could be applied to protect the interests of the individual when sharing tissue and data for research purposes. What this therefore means is that there is a more considered approach in terms of what is necessary to achieve the research aim and what data protection measures could be put in place, rather than a blanket requirement for either consent or anonymisation of data.

The use of safeguards other than anonymisation to ensure data security was referenced in a report published by the Academy of Medical Sciences in 2006 [22]. The report acknowledged that health research will often require access to identifiable information at some stage and moreover, that anonymisation is often not an ‘absolute process’; as there are degrees of anonymisation which depend on the context of any particular situation. This may involve retaining a link to a person’s identity via a unique code (pseudoanonymisation) which means that the data are identifiable to those with legitimate access but ‘anonymised’ to those undertaking research using the data [16]. However, the report published by the Academy of Medical Sciences suggests that a requirement for pseudoanonymisation with a requirement for the researcher to not have access to the key offers minimal security advantage over coded identifiable data sets which are maintained under strict data security policies. This report also reiterates the responsibilities of organisations undertaking research to ensure that they have appropriate data security arrangements in place, something which is now a statutory requirement under the GDPR 2018 requirement for ‘data protection by design and by default’. The GDPR 2018 sets data protection principles which aim to reduce the risk of identifiability, by requiring that secondary processing is not incompatible with the purpose for which it was initially collected, data is limited to what is necessary to achieve the intended purpose, for the minimal time necessary and is processed in a way which ensures appropriate security.^15^ This article suggests that where obtaining consent is not feasible, these safeguards should be considered sufficient to permit the linking of surplus tissue and associated patient data, despite the blanket requirement under the HT Act 2004 that the researcher should not be in possession of information which could identify the person from whom the tissue was removed. This is because the data protection principles sufficiently protect the privacy interests of individuals and furthermore, there is a public interest in enabling research which utilises surplus tissue samples and associated patient data.

To demonstrate this point further, consider a hypothetical research project. A researcher wants to undertake a descriptive study which aims to confirm a histopathological variance in anaplastic thyroid cancer in patients diagnosed with the condition over a 15 year period. Anaplastic thyroid cancer is a rare form of cancer which is fast growing and has often metastasised to other parts of the body before it is diagnosed and therefore survival rates are low [43]. The hypothesis is that the histopathological variance will be identified in patients who had high levels of iron in their blood and lived within a 10 mile radius of a landfill site in the 3 years prior to diagnosis. The researcher has identified 300 thyroid tissue samples which were obtained via routine clinical care with a confirmed diagnosis of anaplastic cancer. The patients were not asked to give their consent for the tissue to be used for research purposes at the time of removal and consent would not now be feasible as many would have died and re-contacting any survivors may not be reasonable due to the time which has passed and the risk of causing distress to any survivors. The researcher aims to obtain and analyse the tissue samples for evidence of histopathological variance and additionally will require information about the person from whom the tissue was removed to confirm blood iron levels and partial postcode to confirm primary residence for the 3 years prior to diagnosis. Furthermore, additional information will also be required to control for confounding factors; the researcher proposes to collect sex, year of birth, smoking status and occupation. These samples and the information would need to be obtained from a number of different NHS hospital trusts and therefore some identifiers would be required to obtain the clinical information and to validate the accuracy of the data; the researchers propose to use the patient’s NHS number and date of birth. Under the ‘consent or anonymise’ interpretation which is currently applied under the HT Act 2004, the researcher would not be able to have access to this information and therefore the research may not be able to proceed because identifiable information is required to locate and link the relevant tissue and clinical data and also certain information which *could* identify the individual is required to answer the research question. This would be regardless of any potential benefit which the research could achieve and therefore the ‘consent or anonymise’ approach acts as a limiter to research which may have public benefit.

If we consider the research project from the perspective of a ‘share and protect’ approach however, then there may be a different outcome. Under this approach, consideration would be given to what data are *necessary* to achieve the research aim and what safeguards would be *appropriate* to protect the interests of the individual. The demographic information, partial postcode, sex, year of birth, smoking status and occupation is required to rule out confounding factors which may otherwise bias the research findings and so is necessary to achieve the research aim. In considering what safeguards could be put in place to protect personal data, a data security policy could be established which imposes limitations in terms of who has access to the data and requires that personal data is kept securely. The NHS number is required to locate tissue and data and the date of birth is required to verify that it relates to the correct person so this data is required for a limited period of time only to achieve a specific function; this data could therefore be replaced with a code once this function has been completed, the key to which is kept securely in a state of limited access. By applying appropriate safeguards which take into consideration the data necessary to achieve the research aim, the potential outcome is that research can be undertaken on surplus tissue with information about the person from whom the tissue came, where obtaining consent for research use would not be feasible, whilst still protecting the interests of those individuals.

## Common Law Duty of Confidentiality

In this article I have considered the relationship between the HT Act 2004 and the GDPR 2018 in relation to whether, in the absence of consent, surplus tissue and data about the person from whom the tissue was removed could be available for research where personal data are necessary to achieve the research aim and applying appropriate safeguards which are broader then just anonymisation. However, the GDPR 2018 also requires that processing of data is lawful, a requirement which is broader than compliance with GDPR 2018 alone, and therefore requires processing to be lawful under other related legislation, as well as the common law duty of confidentiality [35]. Confidentiality is concerned with the control of information which is disclosed within a relationship of trust where there is a reasonable expectation, whether implicit or explicit, that the information will be kept in a state of limited access [44]. Under common law, it is generally considered that where information is given or generated in circumstances where there is a reasonable expectation that a duty of confidence will apply, such information cannot normally be disclosed without the information provider’s consent [45]. Therefore, it is important that the ‘share and protect’ approach would not breach this common law duty of confidentiality. There is no doubt that the information would be considered to be confidential as the information is about a person who could be identified and was disclosed with an expectation that it would be kept confidential. The question therefore remains whether the disclosure itself would be unlawful and in particular, whether safeguards other than anonymisation would be considered sufficient to avoid a breach of the common law. This is a difficult question to answer definitively, primarily due to the fact that the legal basis for a duty of confidentiality and all circumstances under which it is breached remain unclear [46], particularly in a research context as there is no clear legal precedent. However, in the absence of a test case which confirms whether sharing patient information for health research purposes without consent but with safeguards other than anonymisation would be lawful, a clear legal basis could be provided by the Health Research Authority on the advice of the Confidentiality Advisory Group (CAG)^16^ under Sect. 251 of the NHS Act (In England and Wales). The CAG is an independent body established to provide advice in relation to the use of confidential patient information [47]. On the advice of the CAG, the Health Research Authority may permit the common law duty of confidentiality to be temporarily set aside so that personal data can be processed for a defined purpose [47]. This would therefore ensure that processing of confidential information for research purposes remains lawful.

This article has primarily focused on establishing a legal basis for the sharing of surplus tissue samples linked with associated patient data, where obtaining consent would not be feasible. However, there are potentially broader implications of sharing such materials for research purposes within a context of confidentiality and trust which extend beyond ensuring such practices are lawful. Carter et al. [48] reflect on the failure of the care.data initiative, which aimed to extract Primary Care NHS records for purposes beyond direct patient care with an option for patients to opt out, and suggest that whilst there was a legal basis, the initiative failed due to a lack of social acceptance. This resulted in patients losing confidence and trust in what was happening with their data and consequently, significant numbers of people opted out and the initiative was later abandoned [48]. Moreover, whilst 20 years have passed since the publication of the Redfern report detailing the outcome of the inquiry into organ and tissue retention at Alder Hey, the impact which the findings of the inquiry had on trust and confidence [49] will be remembered by many. Therefore, when considering the acceptability of tissue and data sharing for secondary purposes such as health research, acceptability and reasonable expectation are important factors to consider alongside a potential legal basis.

## Conclusion

The legislation regulating human tissue and data in research have developed at different times and have been driven by different motives which has resulted in areas of non-alignment and contradiction, leading to a lack of clarity and the inevitable over caution which often comes with such legal grey areas. Whilst consent undoubtedly plays an important role when undertaking research on tissue and data, it is also important to recognise that due to the processing being secondary, where tissue was removed as part of clinical care and data generated for purposes other than research, consent is not always feasible when the research value is identified at a later point in time. In such circumstances, alternatives to consent which act to safeguard data and respect individual interests should be considered. Furthermore, consistency across different legislation in terms of the acceptability of such safeguards is important to ensure that an effective balance between facilitating research which is in the public interest and respecting and protecting individual rights and interests. Applying the ‘share and protect’ approach which I have set out in this article may help to align the personal data elements of the HT Act 2004 with the data protection requirements of the GDPR 2018. This may better facilitate access to surplus tissue and information which is necessary to achieve the research aim and, by applying appropriate safeguards which involve strict data protection requirements, may help to lessen the requirement for anonymisation to be the only legitimate alternative to consent. However, this is not without its challenges and, due to the wording of the HTA Act 2004 which is clear that in the absence of consent the researcher must not be in possession of information from which the person from whom the tissue came can be identified, a review of the tissue legislation may be required. Whilst the Codes of Practice, published by the Human Tissue Authority as a statutory requirement of the HT Act 2004, are intended to provide practical guidance, set standards and reflect current interpretation of law and regulatory practice, it is unclear whether appropriate safeguards broader then anonymisation would be acceptable under the current wording of the HT Act 2004. Questions also remain with regards to whether applying safeguards, other than anonymisation in the absence of consent where confidential information is shared for research purposes, would leave researchers liable for a claim of breach of confidentiality without Sect. 251 support from the Health Research Authority. Moreover establishing a legal basis for the use of surplus tissue and associated patient information which permits privacy safeguards other than just anonymisation would need to be within the parameters of what patients would reasonably accept and expect to ensure that there is broader social acceptance beyond a legal basis.

## Footnotes

1 Identifiability in this context is considered to be information that may reasonably be expected to identify an individual, alone or in combination with other available information.

2 The Information Commissioners Office recommend undertaking a ‘motivated intruder’ test to assess whether information which is considered to be non identifiable could be used to identify the person to whom the information relates. The ‘motivated intruder’ is taken to be a person who starts without any prior knowledge but who wishes to identify the individual from whose personal data the anonymised data has been derived.

3 Declaration of Helsinki principle 8.

4 The General Data Protection Regulation 2018 repealed the Data Protection Directive 95/46/ec which was enacted into statute in the UK via the Data Protection Act 1998. The GDPR 2018 therefore also repealed the DPA 1998 in the UK which was superseded by the Data Protection Act 2018 to enact national provisions provided for in the GDPR 2018.

5 See Human Tissue Act 2004 explanatory notes, summary and background, paragraph 1.

6 For examples of these discussions, see HC Deb 15 January 2004 col 986 & 993 and HL Deb 22 July 2004 col 377, 378 & 380.

7 Amendment 108.

8 See HC Deb 15 January 2004 col 998.

9 For examples of these discussions see Hansard, Human Tissue Bill House of Commons Debate 15 January 2004 Cols 997, 1003, 1004 & Hansard, Human Tissue Bill House of Lords Debate 22 July 2004 Col 375

10 HL Deb 22 July 2004 Col 427.

11 GDPR Article 6 1 (e) “processing is necessary for the performance of a task carried out in the public interest or in the exercise of official authority vested in the controller”.

12 GDPR Article 9 2 (h) “processing is necessary for the purposes of preventative or occupational medicine, for the assessment of the working capacity of the employee, medical diagnosis, the provision of health or social care or treatment or the management of health or social care systems and services on the basis of Union or Member State law or pursuant to contract with health professionals and subject to the condition and safeguards referred to in paragraph 3”.

13 GDPR Article 7 1–4.

14 GDPR Article 9 1.

15 GDPR Article 5 Sect. 1 sub sections (b), (c) (e) and (f).

16 England and Wales only.
